# Thoracic Ultrasound in Cattle: Methods, Diagnostics, and Prognostics

**DOI:** 10.3390/vetsci12010016

**Published:** 2025-01-02

**Authors:** Luis F. B. B. Feitoza, Brad J. White, Robert L. Larson

**Affiliations:** Beef Cattle Institute, College of Veterinary Medicine, Kansas State University, Manhattan, KS 66506, USA; lffeitoza@vet.k-state.edu (L.F.B.B.F.); rlarson@vet.k-state.edu (R.L.L.)

**Keywords:** bovine respiratory disease, point-of-care ultrasonography, veterinary precision diagnostics

## Abstract

Thoracic ultrasonography (TUS) has emerged as a critical tool in the diagnosis and management of respiratory diseases in cattle, particularly bovine respiratory disease (BRD), which is one of the most economically significant health issues in feedyard operations. The objective of this review is to explore TUS in veterinary medicine, including the historical development, methodologies, and clinical applications for diagnosing and prognosing respiratory diseases. This review also emphasizes the importance of operator training, noting that even novice operators can achieve diagnostic consistency with proper instructions. Ultrasound was introduced in the mid-20th century for back-fat thickness measurements; TUS has evolved to offer a non-invasive, real-time imaging modality that allows for the detection of lung and pleural abnormalities such as consolidations, pleural effusions, and B-lines. These features are vital indicators of respiratory disease, and their early identification through TUS can significantly improve clinical outcomes. Compared to traditional diagnostic methods like auscultation or radiography, TUS provides superior accuracy in detecting both subclinical and advanced respiratory conditions, particularly in high-risk populations. Furthermore, TUS has demonstrated strong prognostic value, with studies showing that the extent of lung consolidation correlates with higher relapse risk, reduced growth performance, and increased mortality.

## Simple Summary

Thoracic ultrasonography (TUS) is an important tool for diagnosing and managing respiratory diseases in cattle, especially bovine respiratory disease, which is a major issue in beef cattle feedyard operations. This review explores how TUS has evolved from its original use in measuring back-fat thickness to becoming a key method for identifying lung problems in cattle, such as pleural effusions and parenchymal lung disease. TUS offers a non-invasive and real-time way to assess pleural space and lungs, helping veterinarians identify early signs of disease. Compared to traditional methods like lung auscultation, TUS is more accurate in finding subclinical or early-stage lung disease, especially in high-risk cattle. It also helps predict which animals are likely to recover or relapse based on the extent of lung damage seen during the scan. Despite its potential, the integration of TUS into routine practice remains limited due to gaps in training and industry awareness. This narrative review highlights recent advancements in TUS, such as improved diagnostic accuracy, faster evaluation protocols, and the ability to predict treatment outcomes. By consolidating current knowledge, this narrative literature review aims to guide veterinarians in adopting TUS for better cattle health management.

## 1. Introduction

Thoracic ultrasound (TUS) in cattle has emerged as a pivotal diagnostic tool, particularly in the assessment of respiratory diseases. This imaging method offers several advantages over traditional bovine respiratory disease (BRD) assessment tools, including its non-invasive nature, lack of ionizing radiation, and ability to provide real-time imaging of thoracic structures [1,2,3,4,5,6]. The application of thoracic ultrasound (TUS) in cattle provides an assessment of diagnostics and prognostics, which can be critical for the accurate management and treatment of bovine health issues [7].

The methodology of thoracic ultrasound in cattle involves the use of low-to-medium-frequency sound waves to allow for thoracic cavity penetration in order to visualize lungs, pleura, and surrounding structures. This technique has been shown to be particularly effective in diagnosing conditions such as bovine respiratory disease (BRD), pleural effusion, diaphragmatic hernias, heart disease, and pericardial involvement in traumatic ruminal pericarditis [6,8,9,10]. Thus, TUS serves as a rapid and practical calf-side diagnostic tool for assessing BRD prevalence and severity, demonstrating a significant correlation with clinical respiratory scores [11,12]. The ability to visualize lung pathology through ultrasound allows for the identification of specific conditions such as pulmonary emphysema and interstitial pulmonary syndrome, with distinct ultrasound artifacts associated with these diseases [9,13,14].

In terms of diagnostics methods, thoracic ultrasound has proven to be superior to traditional radiographic techniques in certain scenarios. For example, Partlow et al. (2017) indicated that TUS is more sensitive than chest radiography for detecting conditions like pneumothorax, but direct comparisons in cattle are less established [15]. Evidence has shown that clinical scores provide limited information for detecting and assessing the severity of respiratory diseases, and an additional diagnostic method like TUS can be applied in combination to improve accuracy [7,16,17]. Increased diagnostic sensitivity and specificity is crucial in chute-side settings where timely diagnosis can significantly impact treatment intervention selection and efficacy. The integration of ultrasound into routine veterinary practice has been advocated as a means to improve diagnostic accuracy and facilitate better clinical decision-making [2,9,10].

Prognostically, thoracic ultrasound provides valuable information that can influence treatment strategies and management decisions. For instance, specific ultrasound findings, such as B-lines, can indicate pulmonary edema and correlate with the severity of respiratory conditions [2,9]. This association allows professionals to assess the prognosis of affected animals more accurately and to optimize treatment plans accordingly.

The TUS method is non-invasive and, when combined with the ability to provide real-time imaging, increases value for veterinarians. The ongoing development of thoracic ultrasound techniques and training programs will likely enhance application in bovine medicine, leading to improved health outcomes and management strategies for cattle. The objective of this narrative literature review is to consolidate the progression and emerging advancements in thoracic ultrasonography for bovine respiratory disease. It highlights gaps in clinical training, recent advancements in portable ultrasonography, and the diagnostic and prognostic potential for field applications, which remain underutilized in current bovine practice. By addressing these aspects, this review provides a contemporary resource for clinicians seeking to implement TUS more effectively.

## 2. Materials and Methods

**Figure 1:**
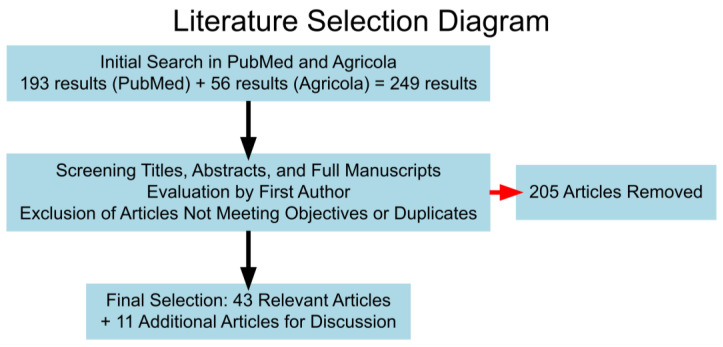
Literature selection diagram illustrating the process of identifying relevant articles for review. Out of 249 initial search results, 207 articles were excluded based on screening criteria (red arrow to the right), resulting in 42 relevant articles. A total of 11 articles were added to enhance the discussion, resulting a final selection of 53 articles.

The databases utilized for the presented narrative literature review include PubMed and Agricola. The key search terms used were ((Ultrasound OR Sonography) AND (Cattle OR Bovine) AND (Thoracic OR Pulmonary) AND (Respiratory OR Pneumonia)). This initial literature search yielded 193 results in PubMed and 56 results in Agricola. The current literature review included original research articles, abstracts, and case reports. Articles were evaluated for inclusion by one author by reviewing the title, abstract, and full manuscript. Articles were excluded that did not meet the study objectives or were duplicated within the two databases utilized. The literature search was conducted for articles published between 1990 and 2023. Of the 249 initial results, 42 documents were included based on relevance to thoracic ultrasonography and bovine respiratory disease, supplemented by 11 additional articles to enhance discussion, totaling 53 final documents, shown in Figure 1.

## 3. Results and Discussion

### 3.1. History

The use of ultrasonography technology in cattle dates back to 1956, when it was utilized for estimating back-fat thickness in live cattle [18]. In 1988, Lamb and associates published an extensive literature review of the veterinary diagnostic ultrasound published work from 1966 to 1986, which yielded 492 articles. Digging deeper into these reports, only five were thoracic ultrasonography-related works, and they were all in equines, ranging from 1981 to 1986 [19]. The earliest manuscript reporting TUS used to detect respiratory disease in cattle (1991) was published in the *Journal of the American Veterinary Medical Association* [1]. The application of TUS in cattle is recent compared to other species. Pivotal published works in the late 1990s and 2000s began to demonstrate the potential of TUS for BRD detection [3,4,20,21,22].

The progression of the raw number of articles reporting TUS over the decades has increased dramatically. A search using PubMed alone yielded 20 publications for the decade of the 1990s, 25 publications for the 2000s, the 2010s had 67 publications, and finally, the 2020s, with only 5 years, have produced 88 publications. Interest in the use of TUS in cattle is rapidly growing.

The early 2000s brought about further advancements, with Jung and Bostedt (2004) exploring thoracic ultrasonography in neonatal calves, identifying lung consolidations and pleural pathologies at stages that traditional methods like auscultation could not detect [21]. Studies began to emerge in the early 2010s that highlighted the efficacy of thoracic ultrasound in diagnosing pulmonary diseases, such as bovine respiratory disease (BRD) [5,6,23,24,25,26]. For instance, a prospective cohort study demonstrated associations between the TUS findings and survival of dairy heifers, underscoring the prognostic value of thoracic ultrasonography [27]. In recent years, TUS for respiratory disease evaluation is being used routinely in dairy operations, specifically in young dairy calves [11,12,13,16,21,28].

The role of thoracic ultrasound in herd health management has evolved alongside advancements in technology. The ability to monitor respiratory health on an individual animal basis has become increasingly important, particularly in high-production cattle operations. This shift towards data-driven veterinary practices to allow for better decisions reflects a broader trend in the industry towards precision livestock farming.

### 3.2. Thoracic Ultrasound Method

Ultrasonography serves as a reliable objective method for diagnosing lung damage, and the TUS findings are well correlated with post-mortem examinations [3,4,7,29]. Using TUS is achievable in a farm setting, with calf-side evaluations being feasible and consistently accurate following proper training [25,26,30,31]. The diagnostic accuracy of TUS for BRD in pre-weaned dairy calves has been reported to have a sensitivity of 79.4% and a specificity of 93.9% for one specific study [26].

**Figure 2:**
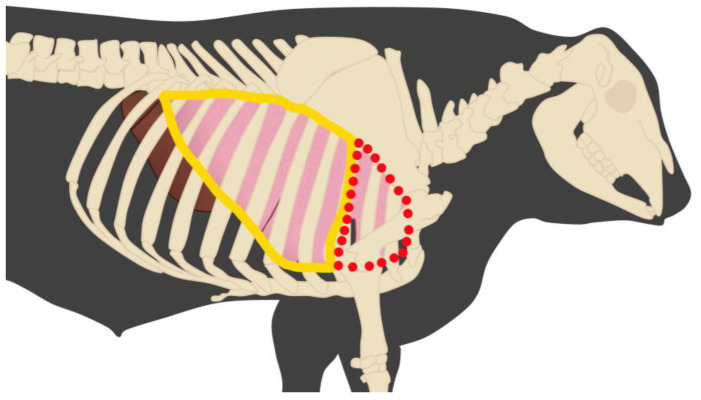
Lateral view of bovine anatomy with respiratory system in evidence. The polygon trace in yellow represents the area of TUS evaluation in an adult bovine (4th to 11th ICS). The area highlighted in red is only possible to be applied TUS in pre-weaned calves; in pre-weaned calves, it is possible to use TUS from the 1st to 11th.

Cattle TUS techniques have evolved significantly over time, with key advancements in probe selection, scanning protocols, and standardization of anatomical landmarks. Typically, a 3.5 to 5 MHz linear or convex probe is used, allowing for adequate penetration for imaging lung structures while maintaining sufficient resolution [24]. Several studies have highlighted the use of intercostal spaces from the 4th to 11th ribs in adult cattle, and pre-weaning calves can be evaluated with TUS starting on the first ICS, as illustrated in Figure 2, which offer the best likelihood for detecting lung and pleural abnormalities [5,9,21,25]. These spaces are examined from dorsal to ventral regions to ensure comprehensive coverage of the lung fields.

Probe orientation is critical; it is typically held parallel to the ribs, with the ultrasound beam perpendicular to the pleural surface to maximize the detection of abnormalities such as consolidation or pleural fluid [14,22]. Buczinski et al. (2015) and Flock (2004) elaborated on the importance of visualizing the pleural line, which provides a key diagnostic marker for lung pathologies, including consolidations and pleuritis [26,29]. Additionally, ultrasonographic methods have been adapted for rapid chute-side assessments, as demonstrated in studies where quick evaluations of lung consolidation were performed in high-risk feedyard cattle, 14 dairy calves [6,11,25,28,31], and veal calves [12,16].

One key advancement in ultrasonographic techniques has been the refinement of a pleural space assessment. Techniques such as “fanning”, where the probe angle is dynamically altered to capture different planes of lung tissue, are commonly used to identify lesions between the ribs [20]. Additionally, coupling agents like isopropyl alcohol are often used in field conditions to optimize probe–skin contact, especially when time constraints prevent extensive preparation, like hair shaving or clipping [6,9,25,30,31,32].

The use of TUS is limited due to logistics at the time and location of examination. Young calves are typically easier to restrain and manipulate the animal position to allow for a thorough examination [25,27]. On the other hand, TUS in adult cattle is more challenging [9,33]. The challenge with ultrasound in adult bovine starts with restraining, that you need at least a head-gate to restrain the animal, and at times a squeeze chute (which may cover the area of interest) [14]. In addition, adult animals are harder to manipulate due to weight and strength, which at times might not allow for a full lung evaluation.

Time constraints are a major limiting factor for the application of thoracic ultrasonography. Published reports have shown that a full TUS evaluation can take from 7 to 45 min [6,9]. While the TUS procedure itself typically takes less than 7 min, the overall time required is often extended by animal handling, which depends on factors such as age, available personnel, and facilities. Improving farm infrastructure to facilitate safe and efficient handling not only enhances welfare but also promotes the safety of veterinarians and workers, ensuring the effective application of TUS. Hence, there is a need to develop new methods to strategically evaluate specific areas of the lung and also specific populations at risk of bovine respiratory disease. Studies such as those by Timsit et al. (2019) highlight the efficiency of chute-side ultrasonography when used strategically, where rapid region-targeted assessments can be completed within minutes (fourth to sixth mid to ventral intercostal spaces), particularly in high-throughput feedyard operations [14]. Another study from Adams and Buczinski (2016) evaluated if a 2 min fixed-time thoracic ultrasound assessment would yield valuable information about dairy calves, and the results showed that a single timepoint 2 min evaluation was associated with health outcomes in these heifers [27]. Another strategic TUS method reported by Pardon (2019) used a simplified scan starting from the caudodorsal tip of the lung to moving cranially in a single motion diagonally up to the fourth intercostal space; this strategic method reduces evaluation time and also provides valuable information about pulmonary health [31].

The balance between speed and accuracy is critical, and in most cases, an extensive and focused examination can be accomplished swiftly without compromising diagnostic accuracy [6,28,34]. The advancements in portable ultrasonography devices and improved operator training have contributed to reducing the time needed for comprehensive examinations, making ultrasound a practical tool for both clinical and field settings.

### 3.3. Respiratory Disease Diagnosis

Normal lung tissue is challenging to visualize via ultrasonography due to air content. Ultrasound waves do not propagate through air; hence, the image of a properly aerated lung is usually dark (low echogenicity), with several pleural line reverberations (artifacts), called A-lines. A-lines appear as echogenic bands parallel to the pleural surface and are consistently observed in a healthy lung. In normal lungs, parietal and visceral pleura often appear as a single, smooth hyperechoic line between the lung tissue and thoracic wall muscles. Lung movement, synchronous with respiration, is normal, and a lack of movement, especially in the pleural line, can indicate pathology (pneumothorax) [22,29].

As respiratory disease develops, there are findings that indicate that the lung and pleura are undergoing injury. The most commonly reported finding is parenchymal consolidation; consolidation refers to non-aerated lung tissue and can manifest in various forms. Hepatization, characterized by lung tissue taking on a liver-like echogenicity, represents one form of consolidation. However, hepatization does not always indicate the most severe lung injury, as it can occur in both active and inactive bronchopneumonia. Inactive bronchopneumonia may present with smaller, localized areas of hepatization. In contrast, fluid alveolograms are typically associated with active bacterial bronchopneumonia, which indicates ongoing inflammation and is generally considered to be more severe. Therefore, the severity of lung injury should be assessed not only by the presence of consolidation but also by the specific characteristics, such as the presence of fluid alveolograms, which denote active disease processes [2,9,22,35]. Consolidation can appear as wedges from the pleural line, nodules, or can affect the entire lung lobe [14,36,37]. Another common abnormal finding is pleural effusion, which is easy to identify as it shows fluid accumulation between the parietal pleura and visceral pleura [22,29]. One artifact usually observed in respiratory disease is the B-line, also called the “comet tail”. This artifact is a vertical beam that often starts at the pleural line and prolongates across the image field [2]. B-lines are usually perceived as a normal artifact if they are rare (2 or less per field). A greater number and width of B-lines are associated with more severe injury [2,38,39,40,41]. Studies consistently show that TUS can detect diseases such as bronchopneumonia, which often manifests as areas of lung consolidation. For example, Buczinski et al. (2014) demonstrated that ultrasound identified lung lesions in 29% of cattle that appeared healthy based on the clinical signs. Furthermore, consolidation depths of more than 3 cm were commonly associated with bronchopneumonia, particularly in cattle suffering from *Mannheimia haemolytica*-induced infections [34]. In addition, bronchopneumonia findings are often found with more severe or developed injury of the cranioventral lobe [42].

The pleura can also present abnormalities. Most common is pleural thickening associated with pleuritis. Another pleural abnormality is called “moth sign”; this abnormality shows an irregular pleural line, with indentations (moth eaten), associated with sub-pleural consolidation [2,3,13,39]. These possible TUS findings or combinations can support respiratory disease detection and prognosis. The use of ultrasound to detect pleural effusion, another common manifestation of BRD, has also been widely documented. Studies by Tharwat et al. (2011), Babkine and Blond (2009), and Masset et al. (2022) confirm the sensitivity of ultrasound for detecting fluid accumulation in the pleural cavity, a feature that is not easily identified using traditional auscultation methods [5,21,22]. In more severe cases of pleuropneumonia, ultrasonography has been instrumental in visualizing both pleural fluid and consolidation that extends deep into the lung tissue [2,3,22]. This is a crucial advancement, as these conditions are typically difficult to diagnose without invasive procedures.

The accurate diagnosis of bovine respiratory disease (BRD) is critical to the effective management and health of cattle, particularly in feedyard operations where BRD is one of the most prevalent and economically significant diseases [43]. The early and precise identification of BRD allows for a timely intervention, reducing the risk of disease progression, relapse, and mortality [36,44]. Accurate diagnosis not only improves animal welfare by preventing chronic illness but also optimizes treatment protocols, reducing the overuse of antimicrobials and enhancing antimicrobial stewardship [44,45]. Additionally, studies like those of Buczinski et al. (2014) have highlighted that ultrasonography can reduce the reliance on empirical treatments by offering a clear picture of the severity of lung involvement [34]. Overall, accurate BRD diagnosis is vital for improving herd health, productivity, and the sustainability of livestock operations [46,47].

The comparative accuracy of ultrasonography versus other diagnostic methods has been a key focus in the literature. For instance, studies by Scott et al. (2013) and Buczinski et al. (2014) indicate that ultrasonography significantly outperforms auscultation and clinical scoring for identifying both subclinical and advanced lung pathology. Auscultation, while a cornerstone of veterinary physical examination, has been shown to miss substantial pulmonary abnormalities, particularly in early disease stages [7,24,34,48]. Furthermore, Buczinski et al. (2015) revealed that ultrasonography offers a higher diagnostic specificity than rectal temperature or respiratory scoring alone, emphasizing its role in confirming suspected BRD cases [26].

### 3.4. Respiratory Disease Prognosis

One of the most promising aspects of thoracic ultrasonography in cattle is its ability to predict outcomes in cattle with respiratory diseases. Numerous studies have highlighted TUS prognostic utility, particularly in identifying cattle that are likely to relapse or experience poor growth performance after treatment [9,14,23,27,36,49,50]. Timsit et al. (2019) showed that cattle with greater lung consolidation depths (≥5 cm) at the first diagnosis of bronchopneumonia had a significantly higher risk of relapse and lower average daily gain (ADG) compared to cattle with less severe lung lesions [14]. This highlights the potential for ultrasonography to guide not only diagnostic decisions but also treatment plans by identifying animals that may require more aggressive or prolonged treatment.

Rademacher et al. (2014) demonstrated that TUS findings can predict mortality risk with significant accuracy. In these studies, large areas of lung consolidation were associated with poor outcomes, including death or culling. Cattle with consolidations exceeding 5 cm were more likely to die or be culled, emphasizing the importance of early detection and the potential to alter treatment protocols based on ultrasound findings [9].

Furthermore, TUS has been shown to be a valuable tool for monitoring treatment efficacy [51]. Serial assessments of lung consolidations during the treatment period provide real-time feedback on whether lesions are resolving or worsening [52]. A study by Wolfger et al. (2015) demonstrated that ultrasound can track the progression or regression of lung lesions, offering an objective measure of treatment success [36]. This capability is particularly useful in field settings, where traditional follow-up methods may be limited by logistical constraints. The ability to monitor animals through non-invasive imaging allows veterinarians to make informed decisions about treatment interventions. This not only optimizes animal health outcomes but also contributes to antimicrobial stewardship by reducing the overuse of antibiotics.

### 3.5. Thoracic Ultrasonography Training

The literature reviewed emphasizes the importance of proper training for veterinarians and technicians performing thoracic ultrasonography in cattle, noting that while the technique can be highly accurate, operator experience plays a crucial role in ensuring diagnostic consistency [6,37,53]. Buczinski et al. (2013) highlight that with adequate training, even novice operators can perform thoracic ultrasonography with consistency and accuracy in detecting lung consolidations and pleural pathologies in calves [4,6,37]. Basic instruction in probe handling and recognition of key anatomical landmarks can allow for less experienced individuals to perform thoracic ultrasound evaluations [29,31,37].

Similarly, Ollivett et al. (2011), Scott (2013), Ollivet and Buczinski (2016), and Pardon (2019) stressed that thoracic ultrasound is not only highly accessible but can be performed efficiently in field conditions given proper training. The training focuses on understanding the placement of the probe in intercostal spaces and interpreting common findings like pleural effusions and lung consolidation, which are critical for diagnosing bovine respiratory disease (BRD) [24,25,31,37]. These studies collectively highlight the necessity of training to maximize the diagnostic potential of TUS, making it a viable and effective tool for diagnosing and managing BRD in cattle.

## 4. Conclusions

In conclusion, thoracic ultrasonography has emerged as an important tool for both diagnosis and prognosis in cattle with respiratory diseases. Its ability to detect subclinical disease, monitor treatment efficacy, and predict long-term outcomes can revolutionize bovine respiratory disease management, particularly in high-producing cattle operations settings where early and accurate diagnosis is critical. The advancements in probe technology, scanning techniques, and point-of-care assessments have made ultrasonography a cornerstone of veterinary diagnostics. As research continues to expand, the use of ultrasonography in bovine respiratory disease will likely become even more practical, offering veterinarians a powerful tool for improving animal health and welfare.

## Data Availability

No new data were created or analyzed in this study. Data sharing is not applicable to this article.
